# DNA methylation of imprinted genes at birth is associated with child weight status at birth, 1 year, and 3 years

**DOI:** 10.1186/s13148-018-0521-0

**Published:** 2018-06-28

**Authors:** Sarah Gonzalez-Nahm, Michelle A. Mendez, Sara E. Benjamin-Neelon, Susan K. Murphy, Vijaya K. Hogan, Diane L. Rowley, Cathrine Hoyo

**Affiliations:** 1 Department of Health, Behavior and Society, Johns Hopkins Bloomberg School of Public Health, 624 N Broadway, Baltimore, MD 21205 USA; 2 Department of Nutrition, Gillings School of Global Public Health, University of North Carolina at Chapel Hill, Chapel Hill, NC USA; 3 Department of Obstetrics and Gynecology, Duke University Medical Center, Durham, NC USA; 4 W.K. Kellogg Foundation, Battle Creek, MI USA; 5 Department of Maternal and Child Health, Gillings School of Global Public Health, University of North Carolina at Chapel Hill, Chapel Hill, NC USA; 6 Department of Biological Sciences, North Carolina State University, Raleigh, NC USA

**Keywords:** DNA methylation, Imprinted genes, Child weight

## Abstract

**Background:**

This study assessed the associations between nine differentially methylated regions (DMRs) of imprinted genes in DNA derived from umbilical cord blood leukocytes in males and females and (1) birth weight for gestational age *z* score, (2) weight-for-length (WFL) *z* score at 1 year, and (3) body mass index (BMI) *z* score at 3 years.

**Methods:**

We conducted multiple linear regression in *n* = 567 infants at birth, *n* = 288 children at 1 year, and *n* = 294 children at 3 years from the Newborn Epigenetics Study (NEST). We stratified by sex and adjusted for race/ethnicity, maternal education, maternal pre-pregnancy BMI, prenatal smoking, maternal age, gestational age, and paternal race. We also conducted analysis restricting to infants not born small for gestational age.

**Results:**

We found an association between higher methylation of the sequences regulating paternally expressed gene 10 (*PEG10*) and anthropometric *z* scores at 1 year (*β* = 0.84; 95% CI = 0.34, 1.33; *p* = 0.001) and 3 years (*β* = 1.03; 95% CI = 0.37, 1.69; *p* value = 0.003) in males only. Higher methylation of the DMR regulating mesoderm-specific transcript (*MEST*) was associated with lower anthropometric *z* scores in females at 1 year (*β* = − 1.03; 95% CI − 1.60, − 0.45; *p* value = 0.001) and 3 years (*β* = − 1.11; 95% CI − 1.98, − 0.24; *p* value = 0.01). These associations persisted when we restricted to infants not born small for gestational age.

**Conclusion:**

Our data support a sex-specific association between altered methylation and weight status in early life. These methylation marks can contribute to the compendium of epigenetically regulated regions detectable at birth, influencing obesity in childhood. Larger studies are required to confirm these findings.

**Electronic supplementary material:**

The online version of this article (10.1186/s13148-018-0521-0) contains supplementary material, which is available to authorized users.

## Background

Understanding factors that influence the risk of obesity in children is crucial to the development of new strategies for obesity prevention. Obesity in early childhood is a risk factor for obesity later in life [1–3] and for a number of chronic diseases in both childhood [4] and adulthood [5]. Birth weight has been associated with weight outcomes later in life, particularly for those who are on the extremes of the birth weight distribution [6–9]. Early identification of obesity or its risk factors will inform interventions to prevent the progression of obesity and its consequences later in life [10]. Consistent with the developmental origins of disease hypothesis, the intrauterine environment is hypothesized to influence an individual’s later susceptibility for chronic diseases [11], including obesity [12, 13].

Epigenetic modifications have been proposed as a mechanism for the in utero origin of later obesity, and a growing literature has found supporting evidence [14–16]. DNA methylation is the most studied epigenetic mechanism in humans, due in part, to its stability. DNA methylation that controls the monoallelic expression of imprinted genes is established during gametogenesis and is stably maintained throughout somatic division [17–21] and therefore provides a stable “register” of early in utero exposures. A study of famine survivors found that adults who experienced famine in utero had hypo-methylation of the imprinted *IGF2* gene compared to their same sex siblings who had not experienced famine in utero [22]*.* The significance of this locus was reported to not have been replicated in this cohort using alternate techniques, including RRBS. However, this technique is generally biased toward CG-rich areas and may not have covered the specific and limited number of CpGs that comprise the IGF2 DMR. Additional genes, such as INSR and CPT1A, have also been identified in association with exposure to the Dutch famine [23]. Another study found that maternal nutrition, affected by striking seasonal variations in food intake in the Gambia, influenced methylation at *RBM46* [24]. A colorectal cancer study found that methylation status of the *IGF2*/*H19* imprinted locus of adult controls was maintained 3 years later [25]. Moreover, a study of NEST children between birth and age 1 year found similar results at the *IGF2/H19* locus [26].

Select imprinted genes have been identified as playing a role in the development of fetal over and undergrowth caused by imprinting defects. The IGF2 locus is used in clinical diagnostic settings to identify Beckwith-Wiedemann syndrome, which is characterized by overgrowth [27], and the H19 locus has been used in the diagnosis of Silver-Russell syndrome (SRS), which is characterized by undergrowth [28].

Although epigenetic data linking DNA methylation and childhood obesity has increased exponentially in the last 5 years [29, 30], few regions agnostically identified have been replicated. This could be in part due to differences in the ethnic composition; however, differences could also be due to the sex composition. At imprinted loci, weight has been associated with the *IGF2* locus. Studies have found a relationship between the *IGF2* domain and fetal growth [31–34] and children’s body composition or weight [32, 35, 36]. Data with directional consistency in associations between additional differentially methylated regions (DMRs) and weight gain are required.

This study aims to assess the association between methylation at nine DMRs of imprinted genes and birth weight for gestational age (BW/GA) *z* score, weight-for-length (WFL) *z* score at 1 year, and BMI *z* score at 3 years. In this analysis, we include the following DMRs: *MEG3* and *MEG3-IG*, which are involved in regulating the delta-like 1 homolog/maternally expressed gene 3 imprinted domain on chromosome 14q32.2; *IGF2* and *H19*, which are involved in the imprinting of the insulin growth factor 2/*H19* domain on chromosome 11p15, which are located upstream of the imprinted promoters of *IGF2* and at the imprinting control region for the *IGF2/H19* imprinted domain near the *H19* promoter, respectively; *PLAGL1* at the pleiomorphic adenoma gene-like 1 locus at 6q24.2; *MEST* at the mesoderm-specific transcript promoter at 7q32.2; *NNAT* at the neuronatin locus at 20q11.23; *PEG3* at the paternally expressed gene 3 promoter region at 19q13.43; and *PEG10* at the epsilon sarcoglycan and paternally expressed gene 10 promoter region at 7q21.3. We selected these regions for their association with infant and child growth [32, 34, 37], chronic disease [22, 38], and parental obesity [39].

## Methods

### Study sample and data collection

We included data from mothers and children in the Newborn Epigenetic Study (NEST). We have described recruitment and enrollment strategies in detail elsewhere [40]. Briefly, between 2009 and 2011, we recruited women from five prenatal clinics and obstetric facilities in Durham, North Carolina. Eligibility criteria included being at least 18 years of age and intention to use one of the qualifying obstetric facilities for delivery. We excluded women if they planned to relinquish custody of the child or planned to move away from the area in the following 3 years. We obtained written informed consent from all participating women. Upon enrollment, mothers completed questionnaires providing information on sociodemographic factors, lifestyle characteristics, and anthropometrics. At delivery, study personnel abstracted birth outcomes from medical records and infant cord blood specimens were obtained to assess offspring methylation. At 1 year, we collected data on child anthropometrics, feeding, and lifestyle. This study was approved by the Institutional Review Board at Duke University Medical Center.

Of the 1700 enrolled, we excluded 396 women for reasons including miscarriage, refusing further participation, moving away from the area, or delivering at a hospital not included in the study. We analyzed DNA methylation data for the first 600 infants in the study. Infants with analyzed DNA methylation were not significantly different than infants whose DNA methylation had been analyzed with respect to race, maternal education, maternal smoking status, maternal pre-pregnancy BMI, maternal age, or weight at age 1 (data not shown).

Among infants with DNA methylation data, birth weight and length measurements were available for 594. At age 1, we used available weight and length measurements for 306 infants, and at age 3, we used available weight and height measurements for 314 children. We calculated BW/GA *z* scores using an international standard [41]. We classified infants with BW/GA below the 10th percentile as small for gestational age (SGA). We calculated WFL *z* scores at age 1 year using WHO standards for children’s exact age [42]. We then calculated BMI *z* scores at age 3 years using CDC standards [43]. We excluded 7 children with a WFL *z* score greater than 5 or less than − 5 at age 1 year, and 11 children with a BMI *z* score greater than 5 or less than − 5. In addition, we excluded infants with possible growth disorders or imprinting defects; therefore, we excluded from analysis infants with DNA methylation values ± 4 standard deviations from the mean (*n* = 6). The current study includes children with available DNA methylation data at birth on at least one of the nine DMRs of interest, and who had plausible length and weight measurements at birth (*n* = 576), age 1 (*n* = 288), or age 3 (*n* = 294). Plausible weight and length was defined as a measurement that fell within the SD limits set for the combined WFL or BMI and that clearly was not a transcript error (e.g., birth weight being copied onto 1-year weight). In addition, we conducted supplemental analysis on 166 children who had non-missing anthropometric values at birth and age 1 and 3 years to assess directional consistency over time.

### DNA methylation

Specimen collection and DNA methylation methods have been described in detail elsewhere [26]. Briefly, we collected infant cord blood specimens at birth. We collected samples in EDTA-containing vacutainer tubes and centrifuged using standard protocols to allow for collection of plasma and buffy coat, with buffy coat used for DNA extraction (Qiagen; Valencia, CA). We stored specimens at − 80 °C until the time of analysis. We extracted DNA using Puregene reagents according to the manufacturer’s protocol (Qiagen) and assessed quantity and quality using a Nanodrop 1000 Spectrophotometer (Thermo Scientific; Wilmington, DE).

We modified infant genomic DNA (800 ng) by treatment with sodium bisulfite using the EZ DNA Methylation kit (Zymo Research; Irvine, CA). Bisulfite treatment of denatured DNA converts all unmethylated cytosines to uracils, leaving methylated cytosines unchanged, allowing for quantitative measurement of cytosine methylation status. We performed pyrosequencing using a PyroMark Q96 MD pyrosequencer (Qiagen). Pyrosequencing assay design, genomic coordinates, assay conditions, and assay validation are described in detail elsewhere [33]. Briefly, we designed assays to query established imprinted gene DMRs using the PyroMark Assay Design Software (Qiagen). We optimized PCR conditions to produce a single, robust amplification product. We used defined mixtures of fully methylated and unmethylated control DNAs to show a linear increase in detection of methylation values as the level of input DNA methylation increased (Pearson *r* is 0.99 for all DMRs). Once we defined optimal conditions, we analyzed each DMR using the same amount of input DNA from each specimen (40 ng, assuming complete recovery following bisulfite modification of 800 ng DNA). We determined percentage of methylation for each CpG cytosine using Pyro Q-CpG software (Qiagen). We performed pyrosequencing assays in duplicate for all specimens whose values fell more than two SD above or below the means, in which case we used the average of the two runs. The values obtained represent the mean methylation for the CpG sites contained within the sequence being analyzed (Additional file 1: Figure S1).

### Statistical analysis

We calculated frequencies and means of sociodemographic variables and conducted multiple linear regression to test the association between DNA methylation and early anthropometric outcomes. We determined covariates a priori based on directed acyclic graphs (DAG). We chose sex as a potential effect measure modifier (EMM), as DNA methylation has been previously shown to vary by sex [44, 45]. We tested the following covariates as potential confounders: maternal education (less than a college degree/college degree or greater), maternal gestational diabetes (yes/no), maternal pre-pregnancy BMI, maternal smoking at any time during pregnancy (yes/no), gestational weight gain, parity (primiparous, multiparous), maternal age at delivery, gestational age, paternal race, maternal race, and date of length and weight measurements relative to child’s birthday. We tested potential confounders in the model one at a time and kept variables if they changed the estimate by more than 10%. Final models included maternal race, maternal education, maternal pre-pregnancy BMI, maternal smoking, maternal age, gestational age, and paternal race. As infants who are SGA may have different growth patterns compared to infants who are average for gestational age, we conducted supplemental analysis to determine the effect of excluding infants who were SGA. We also conducted supplemental analysis including maternal alcohol consumption during pregnancy as a covariate, and an additional supplemental analysis, in which we stratified by race/ethnicity to determine possible effect measure modification.

Previously reported Cronbach’s alpha for correlations among methylation values from all CpGs measured at each DMR was > 0.89 [40]; therefore, we used mean DNA methylation values for each DMR. DNA methylation was assessed in tertiles (low, moderate, high), as both higher and lower levels of methylation have been associated with health outcomes, depending on the DMR [39, 40]. Given the expected 50% methylation of imprinted genes, we used the mid tertile of methylation as the referent category. Thus, results represent the child *z* scores associated with high or low methylation compared to “moderate” methylation. We conducted all statistical analysis using SAS 9.4 (SAS Institute, Inc., Cary, NC).

Among infants, 37.0% of mothers were African American, 28.3% were White/Caucasian, and 34.7% were of other races/ethnicities including Hispanic and Asian/Pacific Islander (Table 1). For the 1-year sample, 37.9% of mothers were African American, 30.2% were White, and 31.9% were “other” race. For the 3-year sample, 39.4% of mothers were African American, 28.3% were White, and 32.3% were of other races and ethnicities. In all samples, the majority of women in the study completed less than a college degree (70.8% for newborns, 66.4% for age 1 year, and 68.4% for age 3 years) and reported not smoking at any point during pregnancy (83, 85.5, and 85.3% for newborns, age 1, and age 3, respectively). Approximately half of the newborn sample reported some sort of alcohol consumption in early pregnancy (50.9%). The mean (SD) maternal age for women in the birth sample was 28 years (± 5.7). Mothers in the 1-year sample were on average 28.0 (± 5.8) years, and those in the 3-year sample were on average 28.1 (± 5.8) years. The mean maternal pre-pregnancy BMI for women in the birth weight sample was 27.4 (± 7.2), BMI for mothers in the 1-year sample was 28.0, and BMI for mothers in the 3-year sample was 28.1 (± 5.8) years. The mean gestational age for the sample at birth was 38.7 (1.7) weeks. The mean birthweight of infants in the sample was 3304 g (± 540). There were no significant differences in the study sample demographic makeup between children or mothers in the newborn, age 1 year, or age 3 year samples (data not shown). However, women were more likely to be college educated in the sample of 166 complete cases, in which children had anthropometric data for all 3 time points.

**Table 1:** Sociodemographic characteristics of study sample

	Newborn	1 year	3 years	Complete cases
Birth weight (grams), mean (SD)	3304.2 (540.3)	–	–	3279.8 (621.1)
Child BMI (kg), mean (SD)	–		16.4 (2.1)	16.4 (1.9)
Birth weight for gestational age *z* score, mean (SD)	− 0.09 (1.0)	–	–	− 0.01 (0.9)
BMI *z* score, mean (SD)	–	–	0.15 (1.3)	0.20 (1.5)
Weight-for-length *z* score, mean (SD)	–	0.83 (1.9)	–	0.78 (1.3)
Race, *N* (%)				
Black	213 (37)	109 (37.9)	117 (39.4)	66 (39.8)
White	163 (28.3)	87 (30.2)	84 (28.3)	44 (26.5)
Other	200 (34.7)	92 (31.9)	96 (32.3)	56 (33.7)
Maternal education, *N* (%)				
Less than HS	175 (32.1)	86 (30.1)	87 (29.9)	48 (29.1)
Completed high school	211 (38.7)	104 (36.3)	112 (38.5)	61 (37.0)
Completed college	159 (29.2)	96 (33.6)	92 (31.6)	56 (33.9)
Missing	31	2	6	1
Maternal smoking, *N* (%)				
Yes	91 (17)	41 (14.5)	42 (14.7)	23 (14.2)
No	445 (83)	242 (85.5)	243 (85.3)	139 (85.8)
Missing	40	5	12	4
Maternal alcohol consumption, *N* (%)				
Yes	179 (50.9)	105 (54.4)	98 (51.0)	63 (57.3)
No	173 (49.1)	88 (45.6)	94 (49.0)	64 (42.7)
Missing	224	95	104	56
Maternal age, mean (SD)	27.8 (5.8)	28.0 (5.8)	28.1 (5.8)	27.9 (5.6)
Maternal pre-pregnancy BMI, mean (SD)	27.6 (7.2)	27.1 (6.6)	27.4 (6.9)	27.2 (6.6)
Gestational age	38.7 (1.7)	38.5 (2.0)	38.6 (1.9)	38.7 (2.1)
Infant sex, *N* (%)				
Male	299 (52.1)	149 (51.7)	152 (51.2)	86 (51.8)
Female	275 (47.9)	139 (48.3)	145 (48.8)	80 (48.2)

## Results

### Birth weight and DNA methylation by sex

In girls, we observed a statistically significant association between high methylation at MEST and greater birth weight for gestational age (*β* = 0.45; 95% CI 0.12, 0.78; *p* value 0.007; data not shown). However, this association did not persist after adjustment. We observed no statistically significant associations between methylation and birth weight for gestational age *z* scores in boys.

### Weight-for-length *z* scores at 1 year and DNA methylation by sex

**Fig. 1:**
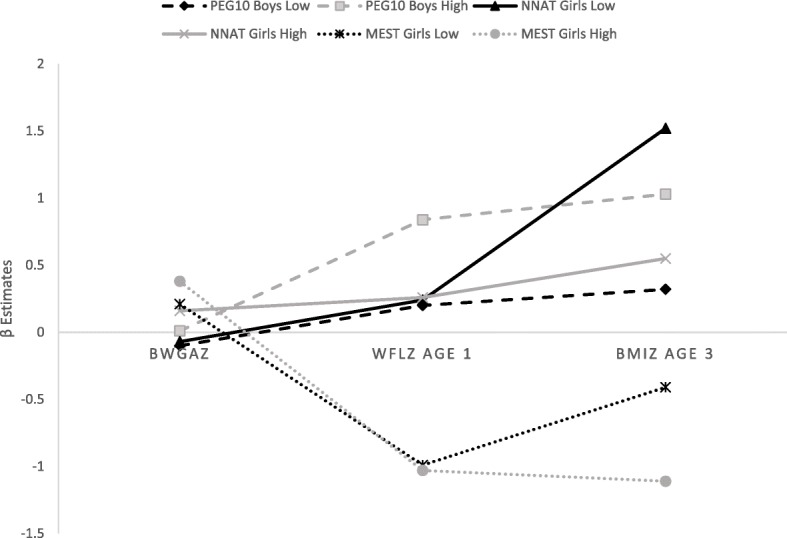
Association between select DMR methylation and BW/GA *z* scores, WFL *z* scores age 1 year, and BMI *z* scores age 3 years. Comparison of girls vs. boys at *PEG10*, *NNAT*, and *MEST* DMRs

After adjustment (Table 2), we observed an association between high *PEG10* DMR methylation and greater WFL *z* scores at 1 year in boys (*β* = 0.84; 95% CI 0.34, 1.33; *p* value = 0.001). Alternatively, low methylation at *IGF2* DMR was associated with a lower WFL *z* score at 1 year in boys (*β* = − 0.63; 95% CI − 1.16, − 0.10; *p* value = 0.02). In girls, both low and high methylation at the *PLAGL1* DMR (low: *β* = 0.72; 95% CI − 1.19, − 0.25; *p* value = 0.003; high: *β* = − 0.81; 95% CI − 1.29, − 0.33; *p* value = 0.0001) and the *MEST* DMR (low: *β* = − 0.99; 95% CI − 1.59, − 0.39; *p* value = 0.002; high: *β* = − 1.03; 95% CI − 1.60, − 0.45; *p* value = 0.001) were associated with lower WFL *z* scores at age 1 year after adjustment (Fig. 1).

**Table 2:** Adjusted results of the association between DNA methylation at birth and birth weight for gestational age *z* scores, BMI *z* scores at age 1 and age 3

	Birth weight for gestational age		WFL age 1		BMI age 3	
	Boys	Girls	Boys	Girls	Boys	Girls
	*β* (95% CI)	*β* (95% CI)	*β* (95% CI)	*β* (95% CI)	*β* (95% CI)	*β* (95% CI)
*MEG3*						
Low	0.09 (− 0.23, 0.40)	0.10 (− 0.22, 0.42)	− 0.67 (− 1.23, − 0.11)	− 0.16 (− 0.70, 0.38)	0.11 (− 0.58, 0.80)	− 0.21 (− 1.04, 0.61)
High	− 0.05 (− 0.35, 0.26)	− 0.26 (− 0.56, 0.04)	− 0.79 (− 1.37, − 0.21)	− 0.08 (− 0.58, 0.42)	0.05 (− 0.64, 0.74)	− 0.29 (− 1.10, 0.52)
*PLAGL1*						
Low	0.20 (− 0.08, 0.49)	0.24 (− 0.06, 0.54)	0.02 (− 0.50, 0.53)	− 0.72** (− 1.19, − 0.25)	0.12 (− 0.53, 0.76)	0.39 (− 1.40, 1.19)
High	0.13 (− 0.16, 0.43)	0.14 (− 0.16, 0.44)	0.52 (− 0.003, 1.04)	− 0.81** (− 1.29, − 0.33)	0.31 (− 0.32, 0.93)	− 0.07 (− 0.81, 0.67)
*PEG10*						
Low	− 0.10 (− 0.39, 0.20)	0.31 (− 0.003, 0.63)	0.20 (− 0.31, 0.71)	− 0.03 (− 0.59, 0.52)	0.32 (− 0.32, 0.95)	0.14 (− 0.70, 0.97)
High	0.01 (− 0.31, 0.32)	0.09 (− 0.24, 0.43)	0.84** (0.34, 1.33)	0.03 (− 0.51, 0.58)	1.03** (0.37, 1.69)	0.04 (− 0.82, 0.89)
*IGF2*						
Low	− 0.15 (− 0.46, 0.16)	0.40 (0.08, 0.72)	− 0.63* (− 1.16, − 0.10)	0.24 (− 0.28, 0.75)	− 0.12 (− 0.76, 0.52)	− 0.10 (− 0.87, 0.66)
High	0.06 (− 0.26, 0.38)	0.21 (− 0.11, 0.53)	− 0.21 (− 0.73, 0.31)	− 0.03 (− 0.53, 0.48)	− 0.14 (− 0.80, 0.52)	− 0.61 (− 1.39, 0.18)
*MEST*						
Low	0.04 (− 0.27, 0.34)	0.21 (− 0.12, 0.34)	0.07 (− 0.47, 0.61)	− 0.99** (− 1.59, − 0.39)	0.26 (− 0.43, 0.94)	− 0.41 (− 1.28, 0.45)
High	− 0.04 (− 0.26, 0.38)	0.38 (0.04, 0.72)	0.32 (− 0.23, 0.86)	− 1.03** (− 1.60, − 0.45)	0.08 (− 0.63, 0.79)	− 1.11* (− 1.98, − 0.24)
*MEG3-IG*						
Low	− 0.01 (− 0.31, 0.34)	− 0.07 (− 0.41, 0.27)	0.09 (− 0.47, 0.65)	− 0.56 (− 1.13, 0.02)	− 0.24(− 0.92, 0.45)	− 0.62 (− 1.44, 0.21)
High	− 0.31 (− 0.66, 0.05)	− 0.08 (− 0.42, 0.26)	− 0.44 (− 1.00, 0.12)	− 0.30 (− 0.83, 0.23)	− 0.10 (− 0.87, 0.67)	− 0.80 (− 1.68, 0.08)
*H19*						
Low	0.29 (−0.04, 0.62)	0.04 (− 0.26, 0.35)	0.26 (− 0.29, 0.80)	− 0.13 (− 0.63, 0.37)	0.24 (− 0.48, 0.96)	0.09 (− 0.73, 0.90)
High	0.03 (− 0.30, 0.36)	0.22 (− 0.09, 0.54)	0.29 (− 0.25, 0.82)	− 0.51 (− 1.02, 0.01)	0.39 (− 0.30, 1.09)	0.34 (− 0.50, 1.19)
*NNAT*						
Low	− 0.06 (− 0.40, 0.28)	− 0.07 (− 0.41, 0.28)	− 0.28 (− 0.89, 0.34)	0.24 (− 0.25, 0.72)	− 0.26 (− 1.00, 0.48)	1.52** (0.69, 2.34)
High	− 0.07 (− 0.42, 0.27)	0.16 (− 0.18, 0.50)	− 0.10 (− 0.69, 0.48)	0.26 (− 0.26, 0.79)	− 0.14 (− 0.82, 0.54)	0.55 (− 0.27, 1.37)
*PEG3*						
Low	0.09 (− 0.19, 0.38)	− 0.09 (− 0.41, 0.23)	− 0.15 (− 0.72, 0.42)	− 0.29 (− 0.84, 0.26)	0.41 (− 0.23, 1.05)	− 0.95* (− 1.80, − 0.10)
High	0.06 (− 0.25, 0.38)	− 0.15 (− 0.48, 0.18)	− 0.20 (− 0.76, 0.37)	− 0.48 (− 1.02, 0.06)	− 0.18 (− 0.91, 0.55)	− 0.46 (− 1.35, 0.44)

Adjusted for maternal and paternal race, maternal education, maternal smoking, maternal pre-pregnancy BMI, maternal age, and gestational age. DNA methylation measured in tertiles, comparing low and high methylation to moderate methylation

*p < 0.05. **Statistically significant after Bonferroni correction (*p* < 0.006)

### BMI *z* scores at 3 years and DNA methylation by sex

At age 3 years, the association between high methylation at the *PEG10* DMR and greater anthropometric *z* score persisted after adjustment in boys (*β* = 1.03; 95% CI 0.37, 1.69; *p* value 0.003). In girls, the association between high methylation at the *MEST* DMR and lower anthropometric *z* score also persisted after adjustment (*β* = − 1.11; 95%CI − 1.98, − 0.24; *p* value 0.01). In addition, we observed an association between low methylation at the *PEG3* DMR and lower BMI *z* score at 3 years after adjustment in girls (*β* = − 0.95; 95% CI − 1.80, − 0.10; *p* value = 0.03).

### Analysis excluding SGA infants

When excluding SGA infants (*n* = 69 at birth, *n* = 31 at 1 year, *n* = 27 at 3 years), additional associations emerged at the *MEST* DMR at birth and the *MEG3*, *H19*, and *NNAT* DMRs at 1 year of age (data not shown). High methylation at the *MEST* DMR was associated with a greater BW/GA *z* score in girls (*β* = 0.32; 95% CI 0.009, 0.63; *p* value = 0.04). At 1 year, high and low methylation at the *MEG3* DMR were associated with a lower WFL *z* score in boys (low: *β* = − 0.81; 95% CI − 1.44, − 0.18; *p* value = 0.01; high: *β* = − 0.91; 95% CI − 1.57, − 0.25; *p* value = 0.008). In girls, high methylation at the *H19* DMR was also associated with a lower WFL *z* score (*β* = − 0.58; 95% CI − 1.15, − 0.008; *p* value = 0.047), and lower methylation at the *NNAT* DMR was associated with a greater WFL *z* score (*β* = 0.63; 95% CI 0.13, 1.13; *p* value = 0.02). All other associations, with the exception of the association between low methylation at the *IGF2* DMR and a lower WFL *z* score at 1 year, persisted after exclusion of SGA infants.

### Analysis including maternal alcohol consumption as a covariate

We conducted additional analyses, in which we included maternal alcohol consumption during early pregnancy as a covariate. We found that all associations between methylation and WFL *z* scores at 1 year remained statistically significant compared to our main analysis (data not shown). Additionally, we observed an association between low IGF2 methylation and greater birth weight for gestational age *z* score in girls (*β* = 0.53; 95% CI 0.13, 0.93; *p* value = 0.01) and an association between both low and high *MEST* DMR methylation and lower BMI *z* scores at 3 years in girls (low: *β* = − 1.31; 95% CI − 2.46, − 0.15; *p* value = 0.03; high: *β* = − 1.40; 95% CI − 2.51, − 0.29; *p* value = 0.01).

### Analysis stratified by race/ethnicity

Additionally, we conducted supplemental stratified analyses to see if there were differences by race/ethnicity, as previous studies have found differential methylation in association with race/ethnicity. The only statistically significant association we observed was between low methylation at the *MEST* DMR and greater birth weight for gestational *z* scores among Blacks (*β* = 0.54; 95% CI 0.09, 0.99; *p* value = 0.02—data not shown). At year 1, we found an association between high *PEG10* DMR methylation and greater WFL *z* scores among Blacks and Whites (Blacks: *β* = 0.69; 95% CI 0.12, 1.27; *p* value = 0.02; Whites: *β* = 0.66; 95% CI 0.11, 1.21; *p* value = 0.02). We also found an association between high *MEG3*-IG DMR methylation and lower WFL *z* scores among Whites (*β* = − 0.64; 95% CI − 1.25, − 0.03; *p* value = 0.04). At 3 years, we found an association between low *MEG3*-IG DMR methylation and lower BMI *z* scores among Blacks (*β* = − 0.76; 95% CI − 1.48, − 0.05; *p* value = 0.04). An association was also observed between low *NNAT* DMR methylation and greater BMI *z* scores among Blacks (*β* = 0.99; 95% CI 0.14, 1.84; *p* value = 0.02).

### Complete case analysis

Supplemental analysis on the 166 infants (*n* = 15 SGA children) who had non-missing anthropometric data for all 3 time points showed directional consistency in all associations. However, not all associations remained statistically significant in the smaller sample (Table 3). In girls, the association between high methylation at the *PLAGL1* DMR and lower WFL *z* scores remained statistically significant (*β* = − 0.82; 95% CI − 1.51, − 0.12; *p* value = 0.02), as did the associations between low and high methylation at the *MEST* DMR and WFL *z* scores (low: *β* = − 1.18; 95% CI − 2.04, − 32; *p* value = 0.009; high: β = − 1.57; 95% CI − 2.4, − 0.73; *p* value = 0.0004). At 3 years in boys, the association between high *PEG10* DMR methylation and greater BMI *z* scores remained statistically significant (*β* = 1.25; 95% CI 0.38, 2.12; *p* value = 0.006). In girls, the association between lower *PEG3* DMR methylation and lower BMI *z* scores at 3 years also remained statistically significant (*β* = − 1.30; 95% CI − 2.34, 0.26; *p* value = 0.02). Notably, additional statistically significant associations were observed between high methylation at the *MEG3*-IG DMR and lower birth weight for gestational age *z* scores in boys (*β* = − 0.77; 95% CI − 1.30, − 0.24; *p* value = 0.005) and between high methylation at the *IGF2* and *PEG10* DMRs and greater birth weight for gestational age *z* scores in girls (*IGF2*: *β* = 0.58; 95% CI 0.03, 1.12; *p* value = 0.04; *PEG10*: *β* = 0.63; 95% CI 0.07, 1.20; *p* value = 0.03).

**Table 3:** Supplemental analysis: complete cases: adjusted regression of DNA methylation at birth and anthropometric *z* scores

	BW/GA		WFL *z* scores 1 year		BMI *z* scores 3 years	
	Boys	Girls	Boys	Girls	Boys	Girls
	*β* (95% CI)	*β* (95% CI)	*β* (95% CI)	*β* (95% CI)	*β* (95% CI)	*β* (95% CI)
*MEG3*						
Low	0.46 (− 0.08, 0.10)	0.23 (− 0.43, 0.85)	− 0.73 (− 1.56, 0.09)	0.01 (− 0.69, 0.71)	− 0.22 (− 1.26, 0.83)	0.24 (− 0.79, 1.27)
High	0.04 (− 0.53, 0.60)	− 0.02 (− 0.68, 0.63)	− 0.87 (− 1.73, 0.00)	0.47 (− 0.25, 1.18)	− 0.10 (− 1.20, 0.99)	− 0.17 (− 1.22, 0.87)
*PLAGL1*						
Low	0.20 (− 0.29, 0.70)	0.30 (− 0.30, 0.89)	− 0.13 (− 0.89, 0.64)	− 0.46 (− 1.17, 0.25)	0.07 (− 0.82, 0.97)	− 0.04 (− 1.01, 0.94)
High	0.26 (− 0.24, 0.76)	0.13 (− 0.44, 0.71)	0.22 (− 0.53, 0.97)	− 0.82* (− 1.51, − 0.12)	0.82 (− 0.06, 1.70)	0.05 (− 0.90, 1.00)
*PEG10*						
Low	− 0.35 (− 0.86, 0.16)	0.40 (− 0.21, 1.00)	0.06 (− 0.77, 0.88)	0.11 (− 0.69, 0.90)	0.53 (− 0.39, 1.46)	0.54 (− 0.47, 1.56)
High	0.03 (− 0.44, 0.50)	0.63* (0.07, 1.19)	0.65 (− 0.12, 1.42)	0.01 (− 0.73, 0.74)	1.25* (0.38, 2.12)	0.64 (− 0.31, 1.58)
*IGF2*						
Low	− 0.46 (− 1.02, 0.10)	0.21 (− 0.37, 0.78)	− 0.51 (− 1.27, 0.25)	0.30 (− 0.46, 1.06)	− 0.40 (− 1.35, 0.55)	0.40 (− 0.58, 1.38)
High	− 0.17 (− 0.70, 0.36)	0.58* (0.03, 1.12)	− 0.02 (− 0.73, 0.68)	− 0.28 (− 0.99, 0.43)	− 0.36 (− 1.25, 0.53)	− 0.25 (− 1.17, 0.66)
*MEST*						
Low	0.41 (− 0.13, 0.95)	− 0.17 (− 0.85, 0.50)	0.20 (− 0.58, 0.99)	− 1.18* (− 2.03, − 0.32)	0.03 (− 0.98, 1.05)	− 0.36 (− 1.57, 0.86)
High	− 0.05 (− 0.56, 0.47)	0.62 (− 0.04, 1.28)	0.38 (− 0.37, 1.12)	− 1.57** (− 2.41, − 0.74)	− 0.14 (− 1.11, 0.82)	− 1.09 (− 2.27, 0.08)
*MEG3-IG*						
Low	− 0.10 (− 0.59, 0.39)	− 0.58 (− 1.30, 0.13)	− 0.22 (− 0.98, 0.55)	− 0.14 (− 1.03, 0.75)	− 0.60 (− 1.56, 0.36)	− 0.15 (− 1.08, 0.78)
High	− 0.77* (− 1.29, − 0.24)	− 0.10 (− 0.77, 0.56)	− 0.70 (− 1.52, 0.12)	− 0.16 (− 0.98, 0.67)	− 0.19 (− 1.22, 0.83)	− 0.59 (− 1.46, 0.27)
*H19*						
Low	0.21 (− 0.32, 0.73)	− 0.28 (− 0.86, 0.31)	− 0.001 (− 0.77, 0.77)	0.24 (− 0.53, 1.01)	0.53 (− 0.39, 1.46)	0.06 (− 0.97, 1.06)
High	0.01 (− 0.52, 0.53)	0.23 (− 0.33, 0.79)	− 0.001 (− 0.75, 0.75)	− 0.41 (− 1.13, 0.32)	0.45 (− 0.45, 1.35)	− 0.08 (− 1.05, 0.89)
*NNAT*						
Low	0.07 (− 0.48, 0.61)	− 0.41 (− 1.02, 0.20)	− 0.89* (− 1.68, − 0.09)	0.47 (− 0.18, 1.13)	− 0.59 (− 1.60, 0.42)	1.12* (0.09, 2.15)
High	− 0.26 (− 0.76, 0.24)	0.39 (− 0.30, 1.08)	− 0.53 (− 1.27, 0.22)	0.39 (− 0.34, 1.12)	− 0.31 (− 1.25, 0.63)	0.24 (− 0.91, 1.38)
*PEG3*						
Low	0.03 (− 0.52, 0.58)	0.16 (− 0.46, 0.78)	− 0.22 (− 1.07, 0.62)	0.00 (− 0.81, 0.81)	0.46 (− 0.48, 1.40)	− 1.30* (− 2.34, − 0.25)
High	0.06 (− 0.54, 0.65)	0.42 (− 0.20, 1.04)	− 0.26 (− 1.17, 0.66)	− 0.30 (− 1.12, 0.52)	− 0.31 (− 1.33, 0.71)	− 0.86 (− 1.91, 0.20)

Adjusted for maternal and paternal race, maternal education, maternal smoking, maternal pre-pregnancy BMI, maternal age, and gestational age. DNA methylation measured in tertiles, comparing low and high methylation to moderate methylation

*p < 0.05. **Statistically significant after Bonferroni correction (*p* < 0.006)

## Discussion

In these analyses, we examined DNA methylation of nine regulatory regions at birth and anthropometric measures at birth and age 1 and 3 years. No DMR showed a consistent association between methylation and anthropometric *z* scores at all 3 time points explored. Our key findings were that high methylation of the sequences regulating the *PEG10* DMR at birth was associated with a higher age 1-year WFL *z* score and 3-year BMI *z* score in boys, low methylation at the *NNAT* DMR was associated with higher BMI *z* scores at age 3 in girls, and high methylation was associated with lower WFL *z* scores at 1 year and BMI *z* scores at 3 years. These associations persisted after excluding SGA infants. Additional findings included associations between methylation at the *PLAGL1* DMR and WFL *z* scores in girls at age 1, as well as an association at the *IGF2* DMR at age 1 among boys. At age 3, we also observed an association between methylation at the *PEG3* DMR and BMI *z* scores in girls. These results suggest that methylation of imprinted genes at birth is associated with anthropometric measures at ages 1 and 3 years, with the *PEG10* and *NNAT* DMRs potentially indicating an early risk for obesity, and *MEST* potentially indicating a lower risk for obesity.

This study adds to a growing body of epidemiologic evidence on early postnatal growth associated with DNA methylation at birth and suggests that DNA methylation at multiple DMRs may be associated with WFL *z* score at age 1 year and BMI *z* score at age 3 years. *PLAGL1*, *MEST*, *NNAT*, and *PEG10* have been associated with obesity or weight in previous literature. Paternal obesity has been previously associated with reduced *PEG10* transcription in mouse placentas [46]. Our results indicating a higher level of *PEG10* methylation is associated with a greater BMI *z* score show a similar pattern. Previous literature has also shown a possible association between increased *MEST* expression and inhibition of adipogenesis [47]. The results of this study echo these findings, as boys with higher than average methylation levels had lower BMI *z* scores. Methylation at the *MEST*, *NNAT*, and *PEG10* DMRs has also been previously associated with paternal obesity [39], and small and large for gestational age [48, 49]. In addition, the *NNAT* gene has been associated with severe obesity in childhood and adulthood [50]. Higher methylation at *PLAGL1* has also been associated with maternal obesity [39], and fetal and postnatal growth [37]. A previous study found a positive correlation between *PLAGL1* methylation and BMI *z* scores at age 1 year. *PLAGL1* is thought to be an imprint control region [51]; however, the implications of this in relation to a potential role in the risk of early obesity are not yet clear. More research is needed to gain a better understanding of the relationship between child BMI and methylation at these DMRs. Previous literature has supported the role of the *IGF2* DMR in fetal growth [31–34] and birth weight [32, 52], as well as infant weight gain [32, 36] and child adiposity [35]. We observed an association between *IGF2* methylation and lower BMI *z* scores; however, this association was not seen at birth or age 3 years.

We observed sex-specific differences in the associations between DNA methylation at birth and anthropometric *z* scores at ages 1 and 3 years. Sex-specific methylation has been previously observed in relation to nutrition and other environmental exposures [44, 45, 53], as well as in relation to outcomes, such as small for gestational age [54]. However, these studies did not find sex-specific differences in methylation in *PEG10*, *NNAT*, or *MEST*. This study also adds to the growing literature on sex-specific DNA methylation.

We conducted additional analysis to explore the influence of infants who are SGA, as their growth patterns may differ from those of infants who are not SGA, and found that SGA may be associated with DNA methylation. No associations between methylation and BW/GA *z* scores remained significant after SGA exclusion, suggesting that SGA may have been driving these associations. We also found that associations between *PEG3* and BMI *z* scores at both ages 1 and 3 years became statistically significant after exclusion of SGA infants. This suggests that perhaps the association between *PEG3* methylation and SGA is in the opposite direction of the association between *PEG3* methylation and BMI *z* scores for non-SGA infants, thus attenuating the original association. However, a cautious interpretation is warranted, as exclusion of SGA infants also decreased the statistical power, which may have led to unstable estimates. Additional supplemental analysis including only the 166 infants who had non-missing anthropometric values at all 3 time points showed that many of our associations remained statistically significant, including our key findings at the *PEG10* and *NNAT* DMRs. This suggests that these associations were not related to differences in the samples at each time point. Additional associations emerged as statistically significant at *MEST*, *IGF2*, *PEG10*, and *NNAT*; however, these results must be interpreted with caution, as the analysis was underpowered. Similarly, the results of the supplemental analysis including maternal alcohol consumption and the analysis stratified by race/ethnicity should be interpreted with caution, as our sample size was greatly reduced, and estimates may be underpowered.

The direction of the association between higher methylation at *MEST* and *z* scores changed from positive to negative from birth to ages 1 and 3. The reasons for this are unclear; however, it is possible that the modest increase in BW/GA *z* scores at birth, which we found to be associated with a higher level of methylation at *MEST*, is also associated with a greater likelihood of becoming lean as the child grows and becomes more mobile. *MEST* expression has been previously associated with obesity in mice [55]; however, a study in humans found *MEST* to possibly inhibit adipogenesis [47].

This study benefits from an ethnically diverse cohort, and prospectively collected data at multiple time points. This facilitates a better understanding of the timing of methylation with regard to our outcome of interest, weight gain. In addition, the use of BMI *z* scores provides widely accepted estimate of adiposity that accounts for a child’s age. However, it is not without limitations. Our study only included nine DMRs of imprinted genes. Although these genes were chosen because they have been linked to growth or chronic disease, it is possible that important genes have been left out of this study. In addition, our study’s small sample size may have limited our ability to see statistically significant differences associated with DNA methylation among our population. Although our analyses were hypothesis driven, multiple testing is a limitation in this study, as it may increase the possibility that our results are observed by chance. However, many associations remained (*PEG10*, *MEST*, *PLAGL1*, *NNAT*) even after the stringent Bonferroni correction. A final limitation was the use of weight status instead of weight gain. Much of the literature has pointed to weight gain in the first year of life as being associated with later obesity [8]; however, there is some literature indicating high weight status in early childhood as a risk factor for later obesity [56]. It is unclear whether or not these findings are related to later obesity.

In addition, the results of this study show differences in anthropometric *z* scores in association with DNA methylation that is either lower or higher than the “average” methylation of our study sample. Notably, the actual change in continuous methylation associated with our results is likely small (approximately 1%). However, even a 1% change in methylation has been previously shown to result in a doubling or halving of gene expression at these imprint regulatory regions [45]. Not assessing DNA methylation continuously may be a limitation of our study, as it creates a challenge in comparing our results to those of other studies. However, we believe the results presented in this study may be meaningful for public health, as it provides a range of methylation values that may be associated with anthropometric, and possibly even adiposity.

## Conclusions

In summary, our study findings suggest that DNA methylation of the *PLAGL1*, *MEST*, *PEG10*, *and NNAT* DMRs at birth is associated with BMI *z* scores in early childhood and varies by sex. Longitudinal assessment of DNA methylation in these DMRs at older age time points is needed to determine whether or not methylation at these DMRs is associated with obesity later in life. Determining the associations between DNA methylation and early obesity risk is important, as DNA methylation of regulatory regions may serve as markers for the assessment of early obesity risk. However, gaining a better understanding of the exposures that affect methylation at these regions is also important, as exposures that modify methylation of regions that are associated with obesity risk may be a good target for early obesity prevention efforts.

## Additional file

Additional file 1: **Figure S1.** Pyrograms from bisulfite pyrosequencing of representative samples. Example results from three individual cord blood specimens are shown for each DMR. The bisulfite-modified version of the sequence to analyze is shown at the top of each pyrogram, and the actual sequencing output, by base, is shown at the bottom. Pyrosequencing is a sequence-by-synthesis method, and the light generated by the incorporation of each nucleotide (*y* axis) is proportional to the amount of the template present in the reaction. For mononucleotides in the template sequence, a single peak height is generated, but for runs of two or more of the same nucleotide in the sequence, the peak height is proportional to that number. Blue diamonds at the top of peaks are the expected peak height based on the sequence. Orange diamonds in yellow-brown vertical bars are bisulfite controls, where the original sequence contains a non-CpG cytosine that should be fully converted by the bisulfite, and thus, no cytosine signal should be detected at these positions. There are no diamonds above the potentially methylated cytosine positions (CGs), which are represented by a “T” position (for unmethylated cytosine) followed by a “C” position (for methylated cytosines). These positions are marked by the grey vertical bars, above which shows the percent methylation for that position of the sequence. (PDF 350 kb)

## Abbreviations

BMI: Body mass index
BW/GA: Birth weight for gestational age
DMR: Differentially methylated region
IGF2: Insulin-like growth factor 2
MEG3: Maternally expressed gene 3
MEG3-IG: Maternally expressed gene 3—intergenic
MEST: Mesoderm-specific transcript
NNAT: Neuronatin
PEG10: Paternally expressed gene 10
PEG3: Paternally expressed gene 3
PLAGL1: Pleiomorphic adenoma gene-like 1
SGA: Small for gestational age
WFL: Weight-for-length
