# Supplementary material for: DNA methylation of imprinted genes at birth is associated with child weight status at birth, 1 year, and 3 years
Source: Clin Epigenetics. 2018 Jun 28;10:90. doi: 10.1186/s13148-018-0521-0 (PMC6025828; doi:10.1186/s13148-018-0521-0)
Supplement: Supplementary file 1 — Figure S1. Pyrograms from bisulfite pyrosequencing of representative samples. Example results from three individual cord blood specimens are shown for each DMR. The bisulfite-modified version of the sequence to analyze is shown at the top of each pyrogram, and the actual sequencing output, by base, is shown at the bottom. Pyrosequencing is a sequence-by-synthesis method, and the light generated by the incorporation of each nucleotide (y axis) is proportional to the amount of the template present in the reaction. For mononucleotides in the template sequence, a single peak height is generated, but for runs of two or more of the same nucleotide in the sequence, the peak height is proportional to that number. Blue diamonds at the top of peaks are the expected peak height based on the sequence. Orange diamonds in yellow-brown vertical bars are bisulfite controls, where the original sequence contains a non-CpG cytosine that should be fully converted by the bisulfite, and thus, no cytosine signal should be detected at these positions. There are no diamonds above the potentially methylated cytosine positions (CGs), which are represented by a “T” position (for unmethylated cytosine) followed by a “C” position (for methylated cytosines). These positions are marked by the grey vertical bars, above which shows the percent methylation for that position of the sequence. (PDF 350 kb) [file 13148_2018_521_MOESM1_ESM.pdf]

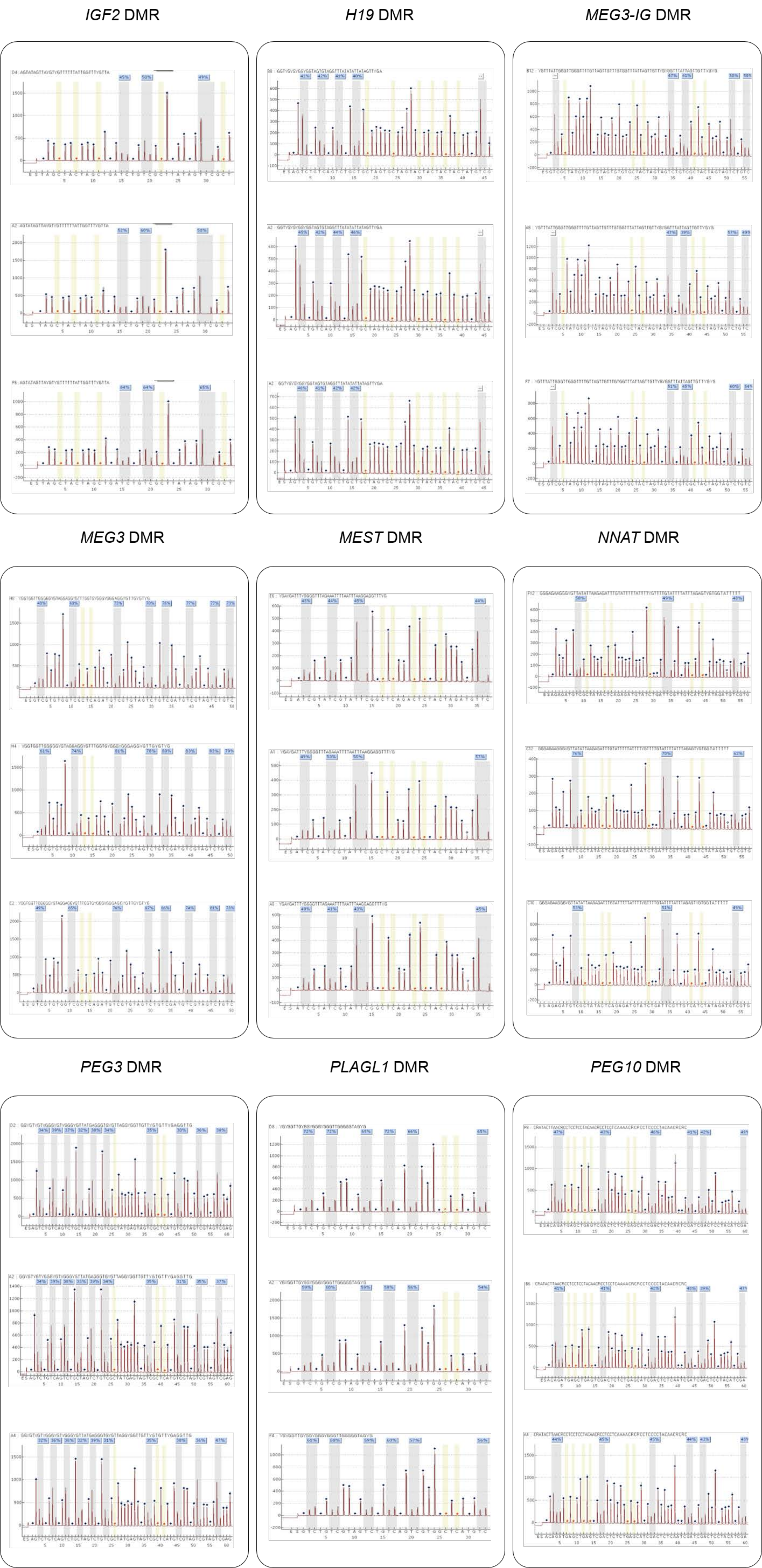
IGF2 DMR
H19 DMR
MEG3-IG DMR
MEG3 DMR
MEST DMR
NNAT DMR
PEG3 DMR
PLAGL1 DMR
PEG10 DMR
